# Temporal hyper-precision of brainstem neurons alters spatial sensitivity of binaural auditory processing with cochlear implants

**DOI:** 10.3389/fnins.2022.1021541

**Published:** 2023-01-04

**Authors:** Michaela Müller, Hongmei Hu, Mathias Dietz, Barbara Beiderbeck, Dardo N. Ferreiro, Michael Pecka

**Affiliations:** ^1^Graduate School of Systemic Neurosciences, Ludwig-Maximilians-Universität, Munich, Germany; ^2^Department of Medical Physics and Acoustics, Carl von Ossietzky University of Oldenburg, Oldenburg, Germany; ^3^Cluster of Excellence “Hearing4All”, Universität Oldenburg, Oldenburg, Germany; ^4^Section of Neurobiology, Faculty of Biology, LMU Biocenter, Ludwig-Maximilians-Universität, Munich, Germany; ^5^Department of General Psychology and Education, Ludwig-Maximilians-Universität, Munich, Germany

**Keywords:** sound localization, hearing, jitter, electrophysiology, computer modeling, electrical hearing

## Abstract

The ability to localize a sound source in complex environments is essential for communication and navigation. Spatial hearing relies predominantly on the comparison of differences in the arrival time of sound between the two ears, the interaural time differences (ITDs). Hearing impairments are highly detrimental to sound localization. While cochlear implants (CIs) have been successful in restoring many crucial hearing capabilities, sound localization *via* ITD detection with bilateral CIs remains poor. The underlying reasons are not well understood. Neuronally, ITD sensitivity is generated by coincidence detection between excitatory and inhibitory inputs from the two ears performed by specialized brainstem neurons. Due to the lack of electrophysiological brainstem recordings during CI stimulation, it is unclear to what extent the apparent deficits are caused by the binaural comparator neurons or arise already on the input level. Here, we use a bottom-up approach to compare response features between electric and acoustic stimulation in an animal model of CI hearing. Conducting extracellular single neuron recordings in gerbils, we find severe hyper-precision and moderate hyper-entrainment of both the excitatory and inhibitory brainstem inputs to the binaural comparator neurons during electrical pulse-train stimulation. This finding establishes conclusively that the binaural processing stage must cope with highly altered input statistics during CI stimulation. To estimate the consequences of these effects on ITD sensitivity, we used a computational model of the auditory brainstem. After tuning the model parameters to match its response properties to our physiological data during either stimulation type, the model predicted that ITD sensitivity to electrical pulses is maintained even for the hyper-precise inputs. However, the model exhibits severely altered spatial sensitivity during electrical stimulation compared to acoustic: while resolution of ITDs near midline was increased, more lateralized adjacent source locations became inseparable. These results directly resemble recent findings in rodent and human CI listeners. Notably, decreasing the phase-locking precision of inputs during electrical stimulation recovered a wider range of separable ITDs. Together, our findings suggest that a central problem underlying the diminished ITD sensitivity in CI users might be the temporal hyper-precision of inputs to the binaural comparator stage induced by electrical stimulation.

## Introduction

**FIGURE 1:**
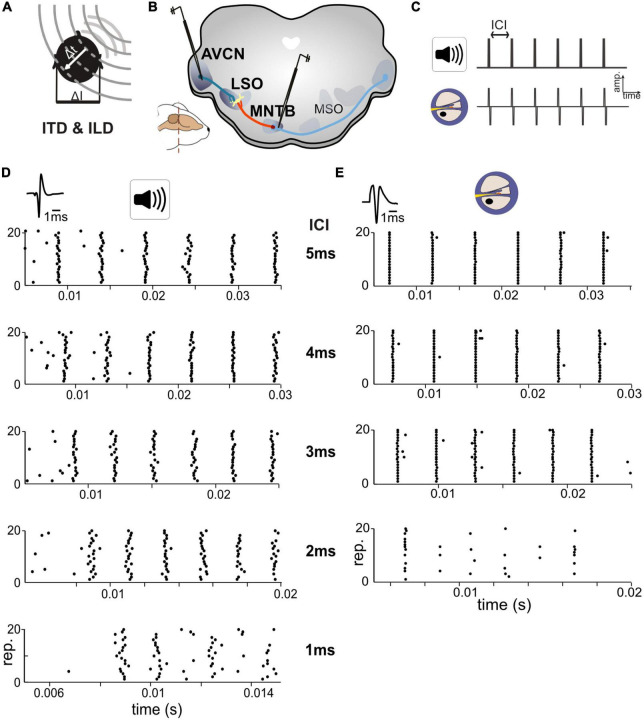
Responses in auditory brainstem neurons differ between acoustic and electrical stimulation. **(A)** The egocentric location of a sound source in the horizontal plane generates specific interaural time differences (ITDs) and interaural level differences (ILDs). **(B)** Extracellular single cell recordings with glass pipettes were conducted in either the antero-ventral cochlear nucleus (AVCN), medial nucleus of the trapezoid body (MNTB), or lateral superior olive (LSO). **(C)** Acoustic and electrical [i.e., cochlear implant (CI)-based] stimulation consisted of a train of six clicks with varying inter-click intervals (ICIs) from 5 to 1 ms. **(D)** Exemplary dot-raster displays of single cell recordings from MNTB [action potential (AP) waveforms shown on top] during acoustic in response to different ICIs. **(E)** Same as panel **(D)** but for electrical stimulation. Each ICI was repeated 20 times. Responses to the individual clicks are readily identifiable, particularly at larger ICIs and overall spike probability decreased with smaller ICIs. Note the difference in spike timing variability (jitter) for each click at all ICIs between acoustic and electrical stimulation. Data from ICI = 1 ms for electrical stimulation was not analyzed due to a strong overlap of APs and electrical artifacts.

Spatial hearing is vital to navigate the busy environments of our daily life. The location of a sound source is neuronally determined by binaural comparison of sound parameters between the two ears, namely interaural time and level differences (ITDs and ILDs, respectively). However, sound localization is impacted already by moderate hearing deficits, resulting in difficulties– amongst others–to orient and identify speakers. In recent years, many efforts have been made to improve hearing based on bilateral cochlear implants (CIs). With CIs, the amplitude envelope of sounds reaching the ears is extracted in multiple spectral channels (Wilson et al., 1991). This envelope information is subsequently passed onto the auditory nerve (AN) fibers by modulating the amplitude of an electrical pulse-train stimulation. The AN fibers then provide input to the brainstem nuclei involved in ILD and ITD detection (see below and Figure 1). While sensitivity to ILDs is rather well maintained in bilateral CI users, ITD sensitivity is very coarse (compared to normal acoustic listeners) and mostly resembles lateralization, even under laboratory conditions (Laback et al., 2015). Moreover, CI-based ITD sensitivity is limited to carrier frequencies of the electrical pulse trains below 500 pulses per second (pps), which is much lower than the typically used pulse rates of CIs pps (Laback et al., 2015). Since ITDs are the crucial cue for human communication and orientation (Wightman and Kistler, 1998; Macpherson and Middlebrooks, 2002), overcoming this lack of electrical ITD sensitivity is desirable. However, the underlying physiological reasons are not well understood. In particular, little is known about potential differences in the neuronal computations between acoustic and electrical ITDs.

Acoustic ITDs are primarily detected by neurons in two brainstem nuclei, the medial superior olive (MSO) and the lateral superior olive (LSO). Since MSO is predominately tuned to low frequencies (<2 kHz), it is regarded to primarily detect fine-structure ITDs (Grothe et al., 2010), while the LSO is mostly sensitive to ITDs in the envelope waveforms of high-frequency carriers (Finlayson and Caspary, 1991). Notably, electrical CI stimulation of AN fibers provides envelope ITD information only and mostly activates higher frequency regions of the cochlea, which predominately innervates LSO. Correspondingly, the observed limits of electrical ITD sensitivity match those of acoustic envelope ITD sensitivity reported for the LSO (Bernstein and Trahiotis, 2002, 2009; Klein-Hennig et al., 2011; Ihlefeld et al., 2014; Laback et al., 2015). Hence, it was recently hypothesized that the LSO is the primary site for ITD sensitivity in CI listeners (Dietz et al., 2016; Hu et al., 2022).

The neuronal processing of ITDs is based on μs precise temporal comparison/integration of synaptic inputs from both ears. Specifically, LSO neurons receive excitatory synaptic innervation from the spherical bushy cells in the ipsilateral antero-ventral cochlear nucleus (AVCN) and inhibitory input from the medial nucleus of the trapezoid body (MNTB) (Grothe et al., 2010). The MNTB itself is innervated by the contralateral AVCN. Both the AVCN and MNTB neurons exhibit faithful locking of their action potential (AP) response times to specific phases of the stimulus waveform (fine structure or envelope transients) (Smith et al., 1998). This enables LSO neurons to generate exquisite ITD sensitivity by detecting differences in input timing. With regard to comparisons to electrical hearing, we have recently demonstrated faithful and very precise ITD sensitivity in high frequency LSO neurons using short acoustic click trains (the stimulus that most resembles electrical CI stimulation) for click-rates up to 500 pps (Beiderbeck et al., 2018). Interestingly, the phase-locking of AN, which directly innervates AVCN (and indirectly the MNTB *via* the AVCN), is severely heightened (e.g., up to approx. a factor of 10 in synchronization index at frequencies < 3 kHz; Dynes and Delgutte, 1992) during electrical stimulation compared to acoustic stimulation. Hence, *a priori*, there is no reason that timing-based processing should be degraded with CIs. However, it is not known to what extent the hyper-precision (phase-locking) found in AN fibers is maintained in the AVCN and MNTB or rather leads to degraded processing, e.g., by failure of transmission. Interestingly, in rats, whose ITD sensitivity is predominately derived from the LSO, lateralization ability to electrical pulse ITDs was shown to be very good (Buck et al., 2021; Rosskothen-Kuhl et al., 2021), suggesting that the LSO could provide behaviorally relevant information during bilateral CI stimulation. Still, it is unclear in what way the information carried by the inputs to LSO, or ITD sensitivity in the LSO itself, is altered during electrical stimulation. More generally speaking, a better understanding of the electrically evoked responses in the brainstem is needed. However, to date an investigation of the LSO pathway during electrical stimulation is missing.

Here, we use a bottom-up approach and ask in what way response features differ between electrical and acoustic stimulation within the initial stages of the LSO circuit. We compare responses to acoustic click and electrical pulses in the AVCN and MNTB by conducting extracellular single neuron recordings in the gerbil, an animal known for good ITD sensitivity (Grothe and Pecka, 2014) both behaviorally and on the level of the LSO. We find that neurons in both of these monaural nuclei exhibit severe hyper-precision and moderate hyper-entrainment during electrical stimulation. This finding establishes conclusively that neuronal ITD detection must cope with highly altered input statistics during CI stimulation compared to acoustic hearing. To determine how ITD computation in LSO might be affected by these changes, we used a previously published model of the LSO circuit that allows using both acoustic and electrical front-ends to compare stimulation types (Ashida et al., 2016, Hu et al., 2022). We carefully adapted the model parameters to match the response characteristics to our physiological recordings in AVCN and MNTB during either stimulation type. The model LSO closely reproduced the ITD sensitivity and its ILD dependency as observed in gerbil LSO. Moreover, the model predicts that ITD sensitivity of LSO to electrical pulses is maintained even for the hyper-precise inputs but exhibits a narrowed dynamic range compared to acoustic stimulation. Calculations of the separability of nearby ITDs suggests heightened resolution at small ITDs during electric compared to acoustic stimulation, while larger ITDs were rendered inseparable. These findings correspond well both with recent reports of high electrical ITD sensitivity in rats, who only experience comparably small ITDs due to the small inter-ear distance (Buck et al., 2021; Rosskothen-Kuhl et al., 2021) as well as with the crude ITD sensitivity found in human CI listeners that often is reduced to lateralization only (Laback et al., 2015). Interestingly, reducing the hyper-precision (phase-locking) of AN fibers during electrical stimulation to acoustically physiological levels recovered a wider dynamic range of ITD coding in the model. Together, our findings suggest that a better understanding of processing of electrical stimuli along the LSO circuit could be crucial for improving ITD sensitivity in bilateral CI users.

## Results

To compare response properties of the excitatory and inhibitory inputs to LSO, we obtained single cell recordings in bushy cells (BCs) in AVCN and principal cells in MNTB (see section “Materials and methods”), in two groups of gerbils (Figures 1A–C). One group of animals was presented with acoustic click-train stimuli (six clicks) at various inter-click intervals (ICIs) between 5 and 1 ms (in 1 ms steps), corresponding to click-frequencies of 200, 250, 333, 500, and 1,000 pps. The second group of animals was implanted unilaterally with an intra-cochlear electrode (see section “Materials and methods”) to allow delivery of electrical stimulation of the AN. As for the acoustic group, the electrical stimuli consisted of a six-pulse long train with ICIs varying between 5 and 1 ms in 1 ms steps. However, strong interferences by the stimulation artifact during recordings prevented analysis of the 1 ms ICI data and only data for ICIs > 1 ms can be presented.

We obtained recordings from 22 AVCN and 18 MNTB neurons (median characteristic frequencies; AVCN: 16.4 kHz, MNTB: 15.0 kHz) during acoustic stimulation, and 11 AVCN and 9 MNTB neurons with electrical stimulation (Figures 1D, E). Stimulus intensity was adjusted for each neuron individually to 30/20 dB above threshold for acoustic and electrical stimuli, respectively (see section “Materials and methods”). We started by analyzing response reliability and timing accuracy of the excitatory and inhibitory inputs to LSO, two crucial factors for the generation of ITD sensitivity. To quantify and compare these parameters for the recorded BC and MNTB neurons, AP responses were analyzed in two ways: first, we determined the “spike probability,” i.e., the average percentage of clicks in the train that elicited APs. For instance, an average response of six APs corresponds to 100% spike probability, as there are six clicks in the train. Second, we calculated the “response jitter,” i.e., the standard deviation of the AP latency relative to the eliciting click (see section “Materials and methods”).

**FIGURE 2:**
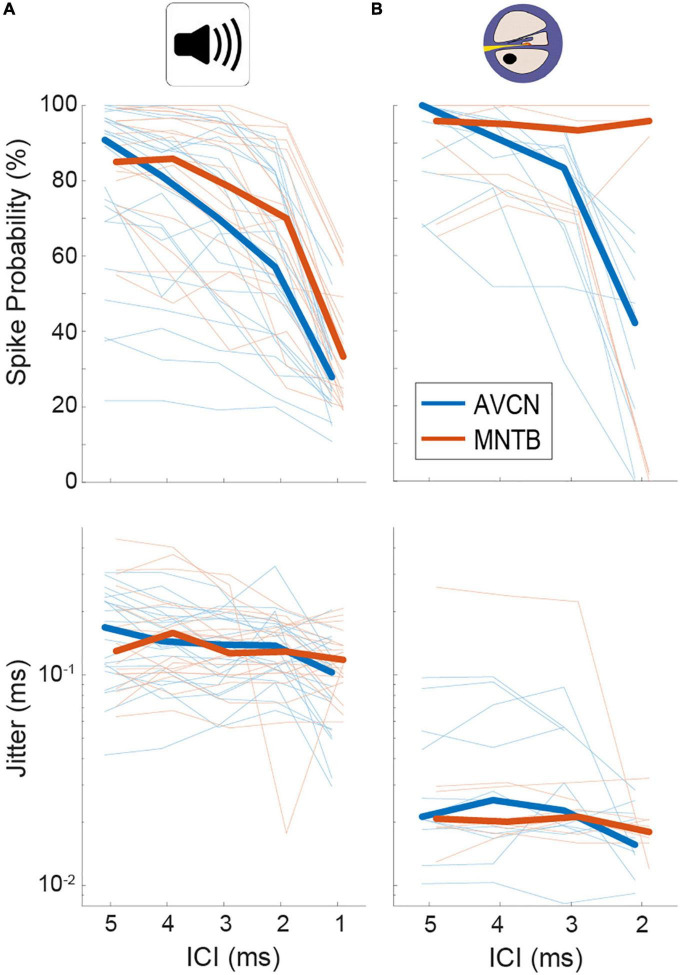
Quantification of spiking probability (upper row) and precision (lower row) in gerbil antero-ventral cochlear nucleus (AVCN) and medial nucleus of the trapezoid body (MNTB) in response to acoustic **(A)** and electrical **(B)** click train stimulation. Plotted are average values for each recorded neuron (thin lines) and the sample medians (bold lines). See also Table 1.

In agreement with previous reports (Joris and Yin, 1998; Smith et al., 1998), we found that spike probabilities in response to acoustic click-trains were similar in BCs and MNTB, with slightly improved spike probabilities found in MNTB at smaller ICIs (Figure 2A and Table 1; *p* = 0.098 Kruskal–Wallis *H*-test). For BCs, the response probability of all recorded neurons dropped considerably at ICI of 2 ms for electrical pulse-trains (Figure 2B). Interestingly, we noted that about half of the MNTB neurons sustained high response probabilities at this IC (Figure 2B), suggesting that at least some MNTB neurons maintain coding capacity at 500 pps. The spike timing jitter was also similar between the two nuclei (Figure 2A and Table 1; *p* = 0.949 Kruskal–Wallis *H*-test). Crucially, electrical pulse-train stimulation resulted in obvious changes in response properties. The spike probability relative to acoustic stimulation tended to be elevated for both BCs and MNTB (Figure 2B and Table 1).

**TABLE 1:** Summary of median values for spike probabilities and jitter in BCs/AVCN and MNTB shown in Figure 2.

		ICI 5 ms	ICI 4 ms	ICI 3 ms	ICI 2 ms	ICI 1 ms
Acoustic: Figure 2A, medians	MNTB (probability)	90.83%	85.42%	76.67%	64.95%	35.88%
	BCs (probability)	84.58%	81.25%	70.00%	57.08%	27.92%
	MNTB (jitter)	0.14 ms	0.15 ms	0.14 ms	0.14 ms	0.11 ms
	BCs (jitter)	0.18 ms	0.14 ms	0.13 ms	0.14 ms	0.10 ms
Electrical: Figure 2B, medians	MNTB (probability)	95.83%	95.00%	93.33%	95.83%	
	AVCN (probability)	100.00%	91.67%	83.33%	42.11%	
	MNTB (jitter)	0.021 ms	0.019 ms	0.023 ms	0.020 ms	
	AVCN (jitter)	0.031 ms	0.025 ms	0.020 ms	0.017 ms	

ICI 5 ms: MNTB *p* = 0.27, BC *p* = 0.058; ICI 4 ms: MNTB *p* = 0.21, BC *p* = 0.089; ICI 3 ms: MNTB *p* = 0.14, BC *p* = 0.61; ICI 2 ms: MNTB *p* = 0.2, BC *p* = 0.017; Mann–Whitney *U*-test.

The largest differences to acoustic stimulation was found in terms of temporal precision, as the jitter during electrical stimulation (Table 1) was approx. 10-fold smaller (Figure 2B and Table 1, all ICIs for MNTB and BCs resulted in *p* < 0.001, Mann–Whitney-*U*-test). These data demonstrate that electrically induced hyper-precision that has been found in AN (Hartmann and Klinke, 1990; Dynes and Delgutte, 1992) is conserved (if not increased) at downstream brainstem nuclei and is likely to influence binaural spatial processing in MSO and LSO.

Yet how exactly could these differences in response properties during electrical stimulation affect spatial processing? For the detection of ITDs, the LSO (as well as the MSO) integrates the inputs from BCs (ipsi-ear) and MNTB (contra-ear) for each click individually. Specifically, using the same acoustic click-train stimuli as for AVCN and MNTB in this study, we had previously determined that LSO neurons exhibit high sensitivity to ITDs of each click in the train, and that the binaural integration of relative strength and timing of inhibition compared to excitation underlies response modulation with changes in ITD in the LSO (Beiderbeck et al., 2018). Hence, we hypothesized that the unusually high precision we found during electrical stimulation for both the excitatory (BCs) and inhibitory (MNTB) input will impact the temporal integration process in the LSO. More generally, we wondered to what extent the observed changes in neuronal responsiveness might constitute a mechanistic explanation for the altered sound localization ability of bilateral CI listeners.

**FIGURE 3:**
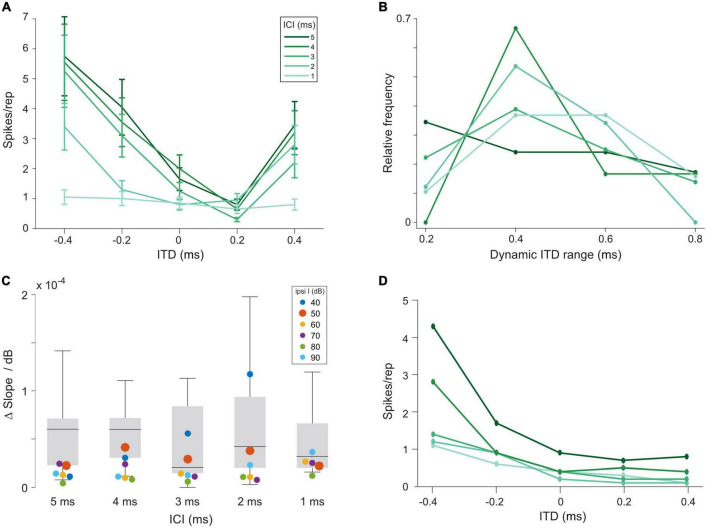
Interaural time differences (ITD) sensitivity of lateral superior olive (LSO) neurons to click trains is maintained over a large range of interaural level differences (ILDs). **(A)** Example rate-ITD function from a gerbil LSO neuron. Plotted are mean rates and the standard errors. **(B)** Histogram of dynamic ITD ranges at ILD = 0 dB across all 15 recorded LSO neurons. **(C)** Quantification of magnitude of changes in the slope steepness of rate-ITD functions with changes in ILD for gerbil LSO (boxplots) and model (at various intensities of the excitatory input, colored dots). The δslope/dB was calculated by first determining the slope of rate-ITD functions (difference in the normalized maximal and minimal spike rates divided by the respective ITD range), and then subtracting these values between the most positive and negative ILD that each LSO neuron was tested for and dividing this difference by the respective difference in ILD. **(D)** Model rate-ITD function during acoustic stimulation (ipsi intensity = 50 dB; ILD = 0 dB).

To this end, we set out to compare ITD sensitivity in the LSO during acoustic and electrical stimulation. We recorded from 15 LSO neurons in response to the same acoustic click-train stimuli used for AVCN and MNTB, presented binaurally at various ITDs (unpublished subset of data reported in Beiderbeck et al., 2018). Typically, response rates were strongly modulated as a function of ITD for a wide range of tested ITDs (Figure 3A). Across the 15 LSO neurons for which we recorded rate-ITD functions at ILD = 0 dB, the dynamic ITD range (range of ITDs between maximal and minimal response rate) reliably covered or exceeded the physiological (i.e., naturally occurring) range of ITD of gerbils (approx. 300 μs, Maki and Furukawa, 2005) for all ICIs (Figure 3B, median dynamic ITD ranges for ICIs of 5, 4, 3, 2, and 1 ms: 400, 400, 400, 400, and 600 μs). This wide range allows for linear attributions of response rates to ITDs, and thus provides the encoding basis for reliable sound source localization based on “hemispheric rate-difference coding,” that is, the encoding of individual ITDs *via* the relative activity levels between the two LSO populations in each brain hemisphere (Klug et al., 2020; for review see Pecka and Encke, 2020). However, ITD sensitivity in the LSO can be influenced by relative changes in the intensity on the two ears (i.e., ILDs, Park et al., 1996; Beiderbeck et al., 2018). To capture this dependency quantitatively in our recordings, we applied various ILDs and determined their effect on the slope steepness of the rate-ITD functions (change in normalized AP rate over the dynamic ITD range, see section “Materials and methods”). On average, the effect of changing ILD had a small to modest effect across all ICIs [Figure 3C, gray bars, medians, and interquartile range (norm. spikes/rep/μs/dB*10^–5^): ICI 5 ms: 6.0, 2.3, 7.1; ICI 4 ms: 6.0, 3.0, 7.1; ICI 3 ms: 2.0, 1.5, 8.4; ICI 2 ms: 4.2, 2.0, 9.4, ICI 1 ms: 3.2, 2.0, 6.6, suggesting that ITD sensitivity in LSO can be maintained over a wide range of binaural conditions.

How does the coding of ITDs change in LSO during electrical stimulation? Unfortunately, recording of single LSO neuron with bilateral electrical CI stimulation proved to be exceedingly difficult. Therefore, to approximate physiological data, we utilized a functional count-comparison model of the LSO (Ashida et al., 2016) as a surrogate for LSO during electrical stimulation. This model can replicate typical response properties of LSO neurons and accompanying spatial perceptions to a wide range of stimulus classes with high precision (Klug et al., 2020; Hu et al., 2022). For example, by connecting this model with an existing acoustically or electrically stimulated AN model, Hu et al. (2022) were able to reproduce most characteristics of acoustically stimulated LSO neurons (Joris and Yin, 1998) and electrically stimulated step-type or trough-type IC neurons (e.g., Smith and Delgutte, 2007, 2008; Chung et al., 2016). Moreover, acoustic and electrical response properties can be readily read-out at various stages of the LSO pathway (Hu et al., 2022). This feature allows comparing model performance to our physiologically recorded AVCN and MNTB data for benchmarking. Specifically, to be able to make informative predictions about changes in ITD sensitivity during electrical stimulation, the model should exhibit similar responsiveness of the excitatory and inhibitory inputs to the LSO during acoustic stimulation. Hence, we determined to what extent the model was able to replicate the response behavior of these LSO inputs.

**FIGURE 4:**
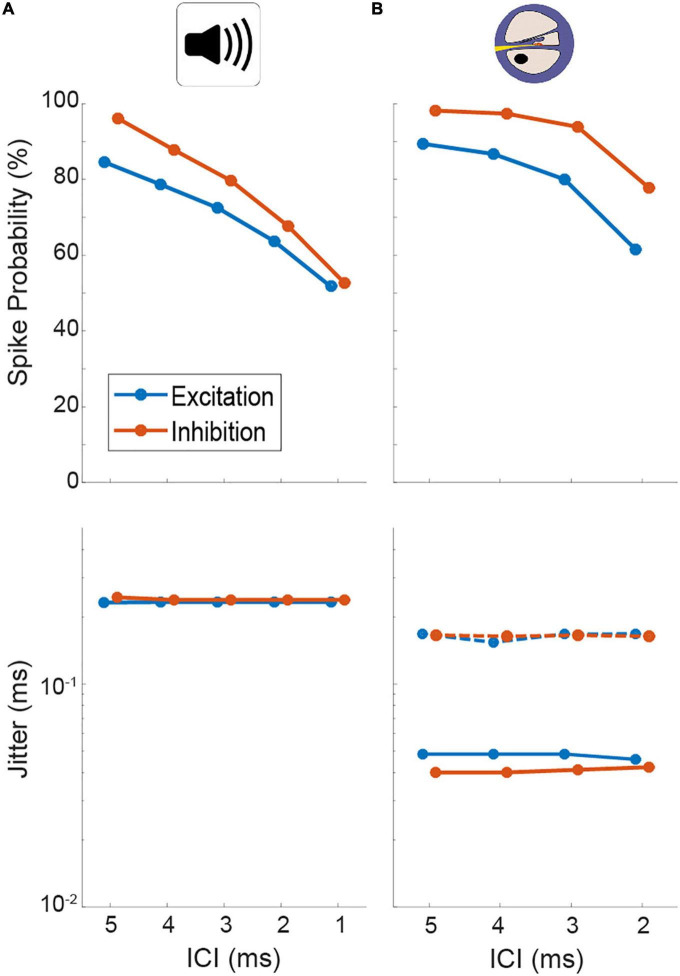
Quantification of spiking probability (upper row) and precision (lower row) of model excitatory and inhibitory inputs to lateral superior olive (LSO) [corresponding to antero-ventral cochlear nucleus (AVCN) and medial nucleus of the trapezoid body (MNTB), respectively] in response to acoustic **(A)** and electrical **(B)** click train stimulation. Plotted are medians and 95% confidence intervals. Dotted lines represent model condition with auditory nerve (AN) jitter levels during electrical stimulation that resemble physiological acoustic jitter levels.

Using previously published general parameter setting (Ashida et al., 2016), we first determined a suitable acoustic stimulus intensity by analyzing the AP responses of the model input stage (AN, note that no explicit CN, and MNTB stage exists in the model, see section “Materials and methods”). At model intensities of 50/60 dB for the excitatory and inhibitory input, the model displayed similar levels of entrainment and jitter as our physiological data (Figure 4A, spike probability for excit./inh.: ICI 5 ms: 84.6/96.1%, ICI 4 ms: 78.6/87.9%, ICI 3 ms: 72.6/79.7%, ICI 2 ms: 63.7/67.8%, ICI 1 ms: 51.9/52.7% and jitter for excit./inh.: 0.23/0.24 ms at all ICIs; compare Figure 2). We further verified that the model also exhibited changes in its responsiveness during electrical stimulation (Figure 4B, spike probability for excit./inh.: ICI 5 ms: 89.4/98.2%, ICI 4 ms: 86.8/97.5%, ICI 3 ms: 80.0/94.0%, ICI 2 ms: 61.6/77.8%) that were comparable to what we had observed experimentally. In particular, the jitter decreased by a similar factor (compare Figure 2) using the electrical front-ends (Figure 4B, excit./inh.: 0.05/0.04 ms at all ICIs).

Based on these identified model parameters we next evaluated the model LSO stage. At 0 dB ILD, the model exhibited ITD tuning with monotonic rate modulation over the range of tested ITDs, resembling the physiological LSO examples qualitatively (Figure 3D). To further test to what extent the model LSO circuit can quantitatively capture the dependency of the slope of ITD tuning on ILD, we applied various ILDs and determined their effect on the slope of the rate-ITD functions (change in AP rate per unit ITD). The previously obtained data set (using the same click-trains) from the gerbil LSO allowed a direct comparison to the model. This comparison demonstrated that the model was adequately capturing the binaural integration process underlying the ITD sensitivity in the LSO. Specifically, the changes in ITD sensitivity with ILD exhibited by the model at 50 dB ipsilateral intensity (Figure 3C, red dots; delta/dB × 10^–5^: ICI 5 ms: 2.3; ICI 4 ms: 4.1; ICI 3 ms: 2.9; ICI 2 ms: 3.8; ICI 1 ms: 2.2) were within the range of observed changes in the gerbil LSO (Figure 3C, gray bars). Hence, these comparisons demonstrate that the response behavior of the model resembles the physiological recordings from gerbil qualitatively and even quantitatively with high accuracy, both for acoustic and electrical pulse-train stimuli. Thus, the model should serve as a valuable proxy for predicting ITD sensitivity of LSO neurons during bilateral electrical stimulation.

**FIGURE 5:**
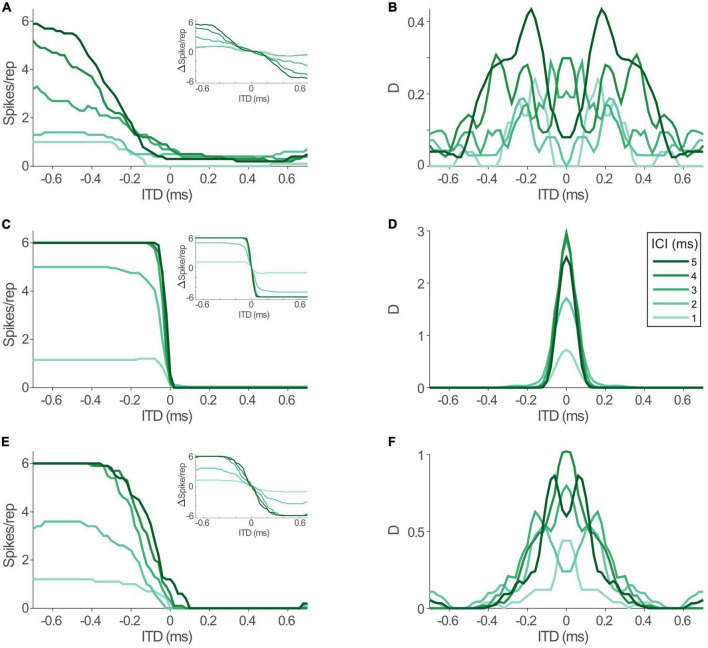
Interaural time differences (ITD) sensitivity in model lateral superior olive (LSO) is maintained during electrical stimulation, but altered by jitter level. **(A)** Model rate-ITD functions during acoustic stimulation. Inset shows hemispheric rate differences for all inter-click intervals (ICIs) (assuming mirrored rate-ITD functions in the LSO on the other brain hemisphere). **(B)** Standard separability D of the hemispheric rate differences for the data shown in panel **(A)**. **(C,D)** Same as in panels **(A,B)**, but for electric stimulation. **(E,F)** Same as in panels **(A,B)**, but for electric stimulation with jitter levels of the model inputs increased to resemble acoustic conditions.

To allow for precise evaluation of changes in ITD coding between acoustic and electric stimulation and its impact on spatial resolution, we determined the informational content of the models’ response toward the ability to distinguish adjacent ITDs with 20 μs increments throughout the entire range of ITDs that is generated by the human head (i.e., inter-ear-distance, approx. ±600 μs, Moore, 2013). To this end we calculated the standard separation “D” (Sakitt, 1973), which quantifies the separability of adjacent ITDs based on the ratio of differences in mean rate and response variability. Since the LSO model is highly deterministic, we used Poisson noise as a conservative assumption (see section “Discussion”). In accordance with the hemispheric rate-difference model of spatial coding (Grothe et al., 2010; Pecka and Encke, 2020), we determined D based on the rate difference between the LSOs on either hemisphere (see insets in Figure 5; responses in one hemisphere were assumed to be mirrored by the LSO in the other hemisphere and subtracted from each other at each ITD). The distribution of D for the rate-ITD functions in response to acoustic stimulation (Figure 5A) spanned almost the entire physiological ITD range of humans (±600 μs) and were either centered on midline (Figure 5B; ICIs of 2, 3, and 4 ms) or peaked at slightly lateralized ITDs (Figure 5B; ICIs of 1 and 5 ms). Next, we tested the model LSO during bilateral electrical stimulation and repeated the D measurements. Notably, ITD sensitivity was maintained for electrical ITDs, as the model LSO displayed steep rate modulation as function of ITD for all ICIs (Figure 5C). However, compared to acoustic ITDs, this modulation (the slope of the function) extended only over a narrower range of ITDs between approx. ±100 and 0 μs ITD, while response rates were effectively identical for more lateralized ITDs to either hemisphere. This “hemispheric binarization” resulted in a highly narrowed distribution of D (Figure 5D), where separability was very high near midline (±100 μs ITD, even higher than for acoustic stimulation) but absent at more lateralized positions on either side. Such an effect can be interpreted as a lateralization effect for human listeners where left can be reliably distinguished from right even for small ITDs around midline, but resolution is almost absent at larger ITDs within each hemisphere. This is contrasted by the more graded distribution of D that we observed during acoustic stimulation, which would allow to also distinguish ITDs within a hemisphere (Figure 5B).

What factors underlie this alteration of the dynamic ITD range? The most drastic change that we revealed for the inputs to LSO during electrical stimulation was the increase in response precision (decrease in jitter). To evaluate the effect of this change in response precision, we re-introduced jitter to the inputs of the LSO model to approximate those of the acoustic stimulation (excit./inh.: 0.17/0.16 ms for all ICIs, dotted lines in Figure 4B). Remarkably, this modification resulted in a re-installment of a wider dynamic ITD range (Figure 5E) and corresponding widening of the range of separable ITDs (Figure 5F). Thus, the model suggests that the increase in spiking precision in response due to artificial electrical stimulation directly alters the ITD coding capacity of LSO during CI-based hearing. Specifically, jitter seems to affect the range of ITDs that alter LSO response rates.

## Discussion

This study is the first to directly assess response properties of brainstem neurons to CI-based stimulation. Spike timing (i.e., jitter) of both BCs and MNTB neurons exhibit severe hyper-precision and moderate hyper-entrainment during electrical stimulation. Thus, the response alterations that have been reported for the AN due to CI stimulation are passed on to subsequent synaptic stages. Specifically, increased response rates and entrainment during electrical stimulation compared to acoustical stimulation had been reported previously at stimulation rates < 800 Hz (Hartmann et al., 1984; van den Honert and Stypulkowski, 1987; Javel and Shepherd, 2000; Litvak et al., 2001). Our recordings in the gerbil AVCN indicate that there seems to be no significant failure of transmission nor compensation caused by the integration mechanisms at the endbulb of Held synapses in BCs for rates below 500 pps. Indeed, precision and spike probability might be further elevated by the coincidence mechanism of the endbulbs. Similarly, the calyx of Held synapse at the MNTB is known to be specialized for maintaining exquisite temporal fidelity and seems to pass on the hyper-precise spiking that is generated by electrical stimulation. Indeed, about half of the MNTB neurons we encountered exhibited high spike probabilities even at 500 pps (ICI = 2 ms), suggesting slightly enhanced fidelity of MNTB compared to BC neurons, albeit not evident in all cells. These observations are significant as they establish conclusively that neuronal ITD detection at the next synaptic stage—MSO and LSO—must cope with highly altered input statistics during CI stimulation compared to acoustic hearing. Thus, any diminished ITD sensitivity on the perceptual level is not caused by a lack of temporal information provided to the binaural integration stage.

This finding naturally raises the questions: which of the two nuclei is the likely site of electrical ITD integration and how do hyper-precise inputs affect the integration mechanism? Perceptual and anatomical data indicate strong bias toward LSO processing (similar cut-off frequencies of ITD sensitivity, basal insertion of electrodes, etc.). Moreover, we had previously established that ITD sensitivity of high-frequency LSO neurons to acoustic click trains is exquisite (Beiderbeck et al., 2018). Therefore, we utilized an established LSO model of acoustic and electrical ITD sensitivity to investigate possible consequences of electrical stimulation. Our data indicate that LSO should not only be able to generate ITD sensitivity to electrical pulses, but be sensitive to the heightened temporal precision, which in turn might be contributing to perceptual alterations of CI users compared to normal listeners. While LSO processing and coding of ITD can be assumed to be similar in all mammals and thus can be extrapolated from gerbils to humans (Grothe and Pecka, 2014), these alterations might have differential effects in large-skulled (e.g., humans) and small-skulled (e.g., gerbils or rats) listeners, because of the difference in the respective available ITD range (approx. ±600 and ±150 μs, respectively). That is, the model predicts that the reported limited perceptual electrical ITD sensitivity in human listeners might not be caused by a general lack of ITD tuning in the brainstem, but rather that the hyper-precise inputs to LSO result in unusually steep rate-ITD functions with altered dynamic range. According to these predictions, the resolution of adjacent source locations might even by improved for small ITDs around midline (up to approx. ±100 μs), while it quickly decreases to inseparability at larger ITDs. Current data on ITD thresholds in CI listeners shows great variability ranging from a few tens μs to >1,000 μs (Laback et al., 2015), but there are no indications of “super-resolution,” i.e., improvements compared to normal hearing threshold. These data are typically collected using a symmetrical forced-choice lateralization paradigm, yet any “super-resolution” area as suggested by our model might not necessarily be located symmetrically at midline, since many factors could shift this region (e.g., electrode mismatch in amplitude or cochlear location, asymmetric de-innervation, subjective training and adaptation effects, etc.). Hence it is currently unclear and would be intriguing to investigate if a small region of improved separability can be found with bilateral CI listeners and/or contributes to the reported variability of thresholds.

However, it must be stressed that the effect of increased separability as measured by D decreases considerably with increasing levels of variability in the LSO population code. Our LSO model is highly deterministic and since we were ignorant about the actual *in vivo* LSO population rate variability, we were required to assume a level. We chose to follow our previous LSO modeling study (Hu et al., 2022) and used Poisson noise as variance measure during the calculation of D. However, this choice might underestimate the true variability *in vivo*, particularly during electric stimulation (Javel and Viemeister, 2000). Importantly, the loss of spatial resolution at larger ITDs is mostly independent of this estimate.

Another notable conclusion of the model’s prediction about improved resolution at small ITDs is that it potentially provides a mechanistic explanation for the recent reports of highly precise ITD sensitivity both on the level of midbrain coding (Buck et al., 2021) and perceptual resolution (Rosskothen-Kuhl et al., 2021) in bilaterally implanted rats. Since these animals have smaller inter-ear-distances compared to human subjects, the predicted region of increased LSO ITD coding capacity would cover a large fraction of the available ITDs in rats, and hence provide an advantageous effect on spatial resolution for almost the entire frontal field. However, as explained above, the extent of the effect is dependent on the actual magnitude of variability in LSO. Our third key finding emphasized the role of jitter on the shape of rate-ITD functions: by increasing the jitter of the inputs to the electrical LSO model to the physiological (i.e., acoustically induced) level, a wider dynamic range of rate-ITD functions (and accompanying separability) was restored. The model consequently suggests that physiological jitter levels are key in establishing the temporal width of the integration window between excitatory and inhibitory inputs. This window width in turn defines how gradually the LSO spike rate is modulated as a function of ITD. In line with this idea, Myoga et al. (2014) demonstrated that jitter affected the interaction of MNTB-derived inhibition and BC-derived excitation and thereby shaped the overall response to specific ITDs. Even though these results were obtained in the MSO, they emphasize the importance of jitter level on the precision of the ITD integration of MTNB and BC inputs and hence the ITD computation mechanism.

Our findings directly extend on previous work on the role of response precision on ITD perception and might provide a complementary mechanistic explanation. Laback and Majdak (2008) proposed that jittering the inter-pulse intervals would lead to improvement of ITD sensitivity by restarting the adaptation process every time the pulse is randomized. They hypothesized that binaural adaption during ongoing high stimulation leads to degraded ITD detection and demonstrated that binaural jitter enhanced ITD sensitivity at higher stimulation rates (≥800 pps). Correspondingly, midbrain recordings showed increased ITD sensitivity by the introduction of short inter-pulse intervals (Hancock et al., 2012; Buechel et al., 2018). Introducing jitter increased firing, presumably by counteracting adaptation to the prolonged stimulation with high-rate click trains. In contrast, our click trains were much shorter compared to earlier studies and resulted in slight increased responsiveness compared to acoustic stimuli and thus might not be directly comparable. Thus, our data indicates that diminished spatial sensitivity might, at least in part, be caused by hyper-precise input timing that results in hyper-acute ITD sensitivity in the LSO. In the future, it would be interesting to repeat our experiments using longer click-trains to test the influence of rate adaptation of the monaural inputs. Moreover, it is currently unclear to what extent the effects of jittering depend on synchrony between the two ears, i.e., our model could be used to test the differential effects of synchronized and desynchronized temporally jittered inputs on electrical ITD processing in LSO.

We used animals with fully developed hearing prior to the experiment. Hence, any experience-dependent mechanisms for the fine-tuning of inputs strength and timing was left in place. Likewise, no degenerative effects had occurred prior to implantation. However, patients that receive CIs typically underwent prolonged periods of deafness and its degradation of the auditory system could lead to a multitude of complications related to this period of inactivity of the system. For example, Hancock et al. (2010) and Tillein et al. (2010) demonstrated reduced ITD sensitivity recorded from the IC and auditory cortex, respectively, in congenital deaf animals. Likewise, it has been shown that the ITD threshold of neonatally deafened animals were similar to the thresholds of normal hearing rats (Buck et al., 2021, Rosskothen-Kuhl et al., 2021), suggesting that electric ITD information can be exploited by the brain. These findings could be explained by our hypothesis that the LSO and not the MSO is mostly responsible for CI-based ITD detection. Excitatory and inhibitory tuning curves of the LSO and the developmental changes of inhibitory projections to the LSO are largely completed before hearing onset (Sanes and Rubel, 1988; Kim and Kandler, 2003). Hence, the LSO circuits develop to functional maturity even in the absence of auditory experience. Any CI-based activity introduced in the system at later stages of life could thus be readily utilized by the LSO circuits to generate spatial sensitivity. In contrast, ITD sensitivity of MSO requires developmental maturation and is dependent on hearing experience in a short critical period after hearing onset (Kapfer et al., 2002; Seidl and Grothe, 2005). Likewise, the data of Buck et al. (2021) and Rosskothen-Kuhl et al. (2021) were obtained in rats, an animal model with high frequency hearing and well-developed LSO (while ITD detection in the MSO can be neglected). In partial support of these rodent data, it has been found that hearing experience in bilateral CI subjects with post-lingual onset of deafness tended to exhibit sizeable ITD sensitivity, while it was not present in subjects with pre-lingual onset of deafness. In contrast, ILD cue sensitivity was similarly present in both groups (Litovsky et al., 2010), supporting our hypothesis of the LSO as the main binaural detector during CI based stimulation. However, electrophysiological recordings of the cellular integration mechanism of the LSO and MSO during CI based stimulation have not been obtained yet. Furthermore, the experimentally obtained rate-ITD functions from the midbrain of implanted rats (Buck et al., 2021) do not readily hint at originating from excitatory-inhibitory interaction and cannot be reproduced by our model.

In summary, our findings suggest that LSO processing is likely the main site of electrical CI-mediated ITD processing and that a key problem underlying the diminished ITD sensitivity in CI users is the temporal hyper-precision of inputs to the binaural comparator stage.

## Materials and methods

All experiments were conducted according to the German animal welfare law (55.2-1-54-2532-53-2015). A total of 37 Mongolian Gerbils (*Meriones unguiculatus*) of either sex and at least 3 month of age were used in this study. After 3 month of age the hearing is fully developed (McFadden et al., 1996), and the cardiovascular system of the animal was capable of enduring long anesthesia (Pecka et al., 2007, 2008, 2010). In animals older than 2 years the hearing threshold and capacity declines, and therefore, animals over 2 years of age were not used in this study (Mills et al., 1990). The animals were kept in Tecniplast Typ 4 cages (610 mm × 435 mm × 215 mm) filled with wooden chipping and wooden wool and a house served for withdrawal. Up to five animals were held in one cage. Temperature and humidity were controlled (temperature 23 ± 2°C, 50 ± 10% humidity) and the animals had a 12-h dark/light circle.

### Anesthesia

Thirty minutes ahead of surgery the animal was injected subcutaneously with an analgesic non-steroidal anti-inflammatory drug (Metacam^®^ 1.5 mg/ml suspension, Boehringer Ingelheim Vetmedica GmbH, Ingelheim, Germany, 0.2 mg/kg). The animal was intraperitoneally injected with 0.5–0.6 ml/100 g body weight Ringer solution mixed with ketamine and xylazine (Ketamine 10%, 100 mg/ml, MEDISTAR GmbH, Ascheberg, 50 mg/kg) und xylazine (Rompun^®^ 2%, 20 mg/ml, Bayer AG, Leverkusen, 2 mg/kg). To maintain anesthesia the animal was injected continuously during the whole experiment with a micropump (Univentor 801 Syringe Pump, Univentor, France) with a flow rate of 1.7 μl/70 g per minute. Besides general anesthesia, local anesthetics were needed for the placing of the head pin as well as for the cochlea implantation. After cutting the skin local anesthetics (Xylocaine^®^ Pumpspray Dental, 50 ml, AstraZeneca GmbH, Wedel, Germany) were used to sedate the muscles and the rest of the surrounding tissue. Surgical anesthesia was reached if the animal did have a negative lid reflex, slight rotation of the *Bulbus*, positive corneal reflex and negative leg withdrawal reflex. Anesthesia was monitored by checking the temperature (*via* a rectal probe), heart rate (EKG), breathing rate, pulse, and oxygen (Pulsoxymeter LifeSense^®^ VET Portable Capnography and Pulse Oximetry Monitor, Nonin Medical, Inc., Plymouth, MA, USA). The body temperature was constantly maintained at 37°C.

### Surgical preparation for cochlea implantation and acute deafening

For the cochlea implantation the post-auricular area was opened with a small incision above the bulla. The skin and the temporalis muscle were removed until the bulla is visible and a bullostomy was performed by drilling a small window inside the bulla. The stapes, stapedial artery, and cochlear fenestra were identified and a small dorsal part of the round window niche was removed to facilitate the insertion of the CI. Afterward the round window membrane was withdrawn in preparation of the deafening of the animal.

For the deafening with neomycin, first the perilymph had to be extracted with a GELoader^®^ (20 μl, Eppendorf SE, Hamburg, Germany). The GELoader^®^ was inserted right at the beginning of the scala tympani. To prevent any destruction of cochlea structures the withdrawal of the perilymph was executed very gently and slowly. Next a careful and slow flushing of the scala tympani with neomycin sulphate (60 mg/ml in NaCl) was conducted. In total the scala tympani was flushed 5 min. This was repeated every 10 min for 90 min. After 90 min the neomycin was extracted. To avoid neurotoxic effects on the spiral ganglia cells the scala tympani was afterward flushed with Ringer solution.

### Auditory brainstem recordings

To test the effectiveness of the deafening procedure on the hearing threshold of the animal an auditory brainstem recordings (ABR) recording was carried out. The animal was placed onto a heating pad powered by an ATC 1000 DC Temperature Controller (World Precision Instruments, Sarasota, FL, USA) in a double-walled sound-attenuated chamber (Industrial Acoustics, GmbH, Niederkrüchten, Germany) lined with acoustic foam. It was set at 37°C to maintain stable body temperature. The reference electrode was inserted subdermally at the vertex, the active electrode was inserted over the bulla and the ground electrode was inserted above the hindlimb. A microphone type 4938 and a preamplifier type 2670 (Bruel and Kjaer, Nærum, Denmark) were used to calibrate the loudspeaker (MF1 Tucker Davis Technologies, Alachua, FL, USA). A short plastic tube was used to extend the loudspeaker. This plastic tube was inserted into the ear. A RZ6 Multi I/O Processor (TDT) was used to generate broadband clicks (0.1 ms duration) and tones of 28, 36, and 44 kHz (5 ms duration, 1 ms rise/fall time) which were produced with Spike software (Brandon Warren, University of Washington, Seattle, WA, USA; pre-amp gain of 20) and presented at a repetition rate of 50 Hz. A RA16 PA 16 Channel Medusa preamplifier (TDT) and RZ6 Multi I/O Processor were used to record ABR waveforms. The recordings were averaged over 1,000 repetitions for each frequency and intensity. If the ABR-based hearing threshold was above 70 dB the deafening procedure was regarded to be successful, otherwise the procedure was repeated.

### Cochlea implantation

After the deafening the cochlea implant (MED-EL animal implants for Guinea pigs and Mongolian gerbils, MED-EL, Innsbruck, Austria) was implanted into the scala tympani of the cochlea. We used bipolar stimulation, where the most apical intra-cochlear electrode was active while the adjacent basal intra-cochlear electrode was used as reference. To ensure the bedding of the cochlea implant throughout the whole recording period a peripheral venous catheter was inserted from the neck muscle to the bulla. The electrode array of the implant was placed within the catheter and lead toward the bulla. After the removal of the catheter, the surrounding muscles kept the implant fixed at this position. The next step was the insertion of the CI. The implant was inserted through the round window and placed within the scala tympani. To ensure the correct insertion depth, the implant was inserted until the black depth marker of the wire. Finally, the implant was fixed with a drop of Histoacryl^®^ and glued to the bulla. This ensured the placing of the cochlea implant within the scala tympani. In all experiments that we had performed the cochlea implant stayed in place.

### Craniotomy and *in vivo* electrophysiology

Recordings for AVCN, MNTB, and LSO were carried out on the same setup using the same hardware. Further details on LSO recordings using acoustic stimuli can be found in Beiderbeck et al. (2018). The animal was placed at a thermostatically controlled heating pad (Fine Science Tools GmbH, Heidelberg, Germany) in a sound-attenuated chamber on a custom-made stereotactic setup. The temperature was monitored using a rectal probe and the head was fixed by a metal rod. The reference electrode was placed in a small craniotomy between bregma and lambda. A second craniotomy and durotomy was drilled behind the sinus transversus lateral to the midline. The lateral position depended on the targeted auditory nuclei. The surface of the brain was covered with physiological NaCl solution (0.9%). To find the correct nuclei the head of the animal was stereotactically aligned.

A glass electrode was lowered into the brain with an angle of 20° by using a motorized micromanipulator (Inchworm controller 8200, EXFO Burleigh Products Group, ON, Canada). APs were recorded using a glass electrode filled with 5 units/μl horseradish peroxidase (HRP) (Sigma-Aldrich Corp., St. Louis, MO, USA) The HRP was diluted in 10% NaCl solution. This resulted in a tip resistance of 8–12 MOhm. The recorded extracellular neuronal signals were pre-amplified (Electro 705, World Precision Instruments, Sarasota, FL, USA), further amplified (TOE 7607, Toellner Electronic, Herdecke, Germany), and filtered (Hum Bug Noise Eliminator, Quest Scientific Instruments Inc., New Delhi, India). A real-time processor (RP2, Tucker Davis Technologies Inc., Alachua, FL, USA) transferred the signal to a computer. Stimulus presentation was controlled in Brain Ware (Jan Schnupp, Tucker Davis Technologies Inc., Alachua, FL, USA) or AudioSpike (Hörtech gGmbH, Oldenburg, Germany) using a sound card interface (Fireface UFX, RME-Audio). Brain Ware and AudioSpike were also used to monitor the recording online and for offline spike sorting.

### Stimulus generation

Two different groups of animals were used: implanted (to record responses in AVCN and MNTB to electrical pulses) and control (to record responses in AVCN and MNTB to acoustic clicks). For the control group, only acoustic stimuli were applied. For the implanted group electrically generated stimuli were applied.

Acoustic stimuli ranging between 0.1 and 90 kHz were generated digitally and altered to an analog signal (RX6, Tucker Davis Technologies Inc., Alachua, FL, USA) at 200 kHz sampling rate, attenuated (PA5; Tucker Davis Technologies Inc., Alachua, FL, USA), and transferred to the headphones (Etymotic ER-4 microPro, Houston, TX, USA or custom-made electrostatic headphones). In a subset of the experiments acoustic stimuli ranging between 0.1 and 90 kHz were generated digitally and sent to an Audio Interface (RME Fireface UFX II, Audio AG, Haimhausen, Germany) at 192 kHz. The audio interface transferred the acoustic stimuli to the headphones (Etymotic ER-4 microPro, Houston, TX, USA). For both auditory nuclei (CN and MNTB) white noise bursts (duration 200 ms; rise/fall times of 5 ms) were presented monaurally. The ipsilateral ear was stimulated for the CN and the contralateral ear was stimulated for MNTB. When a neuron was isolated, its characteristic frequency (CF) and threshold was determined using pure tones at various frequency and intensity combinations. Subsequently, peristimulus time histograms (PSTH) were recorded at CF at 20 dB above threshold. Moreover, the neurons broadband threshold was determined audio-visually using white noise stimuli. Afterward a train of six clicks with a single-click-duration of 50 μs was presented at five different ICIs (ICIs; 5, 4, 3, 2, and 1 ms) in a pseudo-randomized order.

Electrical stimuli were generated digitally, altered to an analog signal (RX6, Tucker Davis Technologies Inc., Alachua, FL, USA) or transferred to an Audio Interface (RME Fireface UFX II, Audio AG, Haimhausen, Germany) and forwarded onto a voltage-current converter (ICS5, Thomas Wulf Elektronik, Frankfurt, Germany) and delivered to the animal *via* the cochlea implant. The voltage used for stimulation varied between 0.2 and 1.2 V. A click train with a single-click-duration of 110 μs (Anodic phase 50 μs, Cathodic phase 50 μs, and Interphase 10 μs) was used at a ICIs of 5 ms as search stimulus. After encountering a neuron, its electrical threshold was determined audio-visually. Next a train of six clicks with a single-click-duration of 110 μs (Anodic phase 50 μs, Cathodic phase 50 μs, and Interphase 10 μs) was presented in a pseudo-randomized order at five different ICIs (5, 4, 3, 2, and 1 ms) 2 dB above threshold.

### Models

The coincidence counting model of LSO was fully described and suggested as a fundamental operation in both ILD-coding and phase-coding of AM sounds in Ashida et al. (2016). Briefly, the model compares the weighted numbers of excitatory and inhibitory inputs within a pre-defined time window and generates an AP when the total number reaches the threshold. More specifically, in the current simulation, the spikes were counted within a coincidence window size of 0.8 ms for the ipsilateral excitatory inputs and an inhibition window size of 1.6 ms for the contralateral inhibitory inputs. If the sum of excitatory spikes (each counting +1), and inhibitory spikes (each counting −2) reaches the response threshold of 8, an AP was generated. Within each refractory period of 1.6 ms, only the first one led to a spike and the others were discarded. The same peripheral models as Hu et al. (2022) were used. Briefly, the periphery model of Bruce et al. (2018) was applied in the same fashion as in Klug et al. (2020) for simulations of acoustic hearing. The AN model parameters were kept unchanged from Zilany et al. (2009, 2014) and Bruce et al. (2018). To simulate electrical hearing, the acoustic auditory periphery model was substituted by the AN model of Hamacher (2004). It includes cell membrane, membrane noise, refractory period, and latency and jitter. Most parameters were same as in Hamacher (2004) and Fredelake and Hohmann (2012), except that the mean and standard deviation of the latency and jitter were adjustable to fit the physiologically recorded AVCN and MNTB data. At the binaural interaction stage, the coincidence-counting LSO model of Ashida et al. (2016) was used. It receives excitatory synaptic inputs from ipsilateral AN fibers and inhibitory inputs from contralateral AN fibers. Different from Klug et al. (2020) and Hu et al. (2022), the default parameters of Ashida et al. (2016) were used with the length of the rectangular excitatory coincidence window and the rectangular inhibitory window (*W*_*inh*_) of 0.8 and 1.6 ms, respectively.

A train of six rectangular clicks with a single-click-duration of 50 μs or six biphasic constant-amplitude pulse trains (cathodic/anodic, 50 μs phase duration) were generated digitally with sample rate of 100 kHz and used as the inputs to the acoustic or electrical AN model, respectively. The results of five ICIs (5, 4, 3, 2, and 1 ms) at different presentation levels over 200 repetitions were obtained for each condition.

### Histology and data analysis

MNTB and BCs were positively identified in most recordings by demonstrating a pre potential (PP) in the wave form. Additionally, HRP was used to identify the recording site of the stimulated cells. By applying 1 μA for 8 min at the end of the experiment HRP was administered iontophoretically through the recording electrode. After the administration of HRP a lethal dose of Narcoren (Pentobarbital 500 mg/kg) was injected intraperitoneally. Firstly the animal was perfused for 10 min with Ringer-solution containing NaCl (0.9%), heparin (100 μl), and 5 mM phosphate-buffered saline (PBS) in H_2_O. Afterward the animal was perfused with 4% paraformaldehyde (PFA in PBS pH 7.4) for another 10–25 min. Finally the brain was removed, placed into 4% PFA for 1–2 days at 4°C, then washed three times for 10 min in PBS (0.02 M) and put into 4% agarose. Using a vibratome the brain was sliced into coronal brain slices of 50–80 μm thickness. A diaminobenzidine (DAB) substrate kit (Vector Laboratories, Inc.) served to stain the brain against HRP. The brain was incubated for 8 min with DAB, then the slices were washed three times for 10 min with aqua dest and PBS and placed onto glass objectives to dry overnight. On the following morning, the slices were counterstained with neutral red solution [1 g neutral red in 4 ml acetate buffer (0.2 M) pH + 4.8 mixed with 100 ml distilled water] and covered with glass objectives slides and DePeX.

Custom-made programs in Matlab (The MathWorks Inc.) were used for data analysis. The physiological data was analyzed for each ICI individually by calculating the median spike probability and jitter for AVCN and MNTB, and dynamic ITD range and delta slope per dB ILD for LSO. The spike probability of each neuron was calculated by dividing the median spike rate per trial by 6 (the number of pulses per trial). The jitter was calculated by the standard deviation of the AP latency relative to the eliciting click. The dynamic ITD range was defined as the range between the ITDs that elicited maximal and minimal spike rates. The effect of ILDs on ITD sensitivity was assessed by calculating the delta slope: the difference in the normalized maximal and minimal spike rates were divided by the respective dynamic ITD range to yield the slope. These values were subtracted between the most positive and negative ILD that each LSO neuron was tested for and divided by the respective difference in ILD. Artifact removal in recordings during electrical stimulation was performed by zeroing the amplitude values of the raw data traces (typically ±10 samples centered on the artifact peak). Spike detection and spike time determination were performed subsequently. A non-parametric ANOVA was used to test for across-group significance. The Mann–Whitney *U*-test served to test for statistical significance of individual ICIs.

The standard separation D is calculated as previously described (Sakitt, 1973):


Dn=|mun+1−mun|/(sqrt(sigman+1*sigman)),

where mu_n+1_ and mu_n_ are the mean values of the hemispheric rate differences to two ITD values while sigma_n+1_ and sigma_n_ are their standard deviation. D_n_ was subsequently smoothed using a 5-sample moving average filter. Sigma of model responses follow a Poisson noise assumption.

## Data availability statement

The raw data supporting the conclusions of this article will be made available by the authors, without undue reservation.

## Ethics statement

This animal study was reviewed and approved by the Ethikkommission der Regierung von Oberbayern.

## Author contributions

MP conceived the study, designed the physiological experiments, and wrote the first draft of the manuscript. MM designed and conducted the cochlear implantations and carried out the experiments with recordings in AVCN and MNTB. BB conducted the LSO recordings. HH and MD generated the model. HH delivered the model results. MM and DNF analyzed the results. MP and DNF designed and generated the figures. MM and HH made contributions. All authors provided comments and approved the manuscript.
